# Liver Tumor Burden in Pancreatic Neuroendocrine Tumors: CT Features and Texture Analysis in the Prediction of Tumor Grade and ^18^F-FDG Uptake

**DOI:** 10.3390/cancers12061486

**Published:** 2020-06-07

**Authors:** Alessandro Beleù, Giulio Rizzo, Riccardo De Robertis, Alessandro Drudi, Gregorio Aluffi, Chiara Longo, Alessandro Sarno, Sara Cingarlini, Paola Capelli, Luca Landoni, Aldo Scarpa, Claudio Bassi, Mirko D’Onofrio

**Affiliations:** 1 Department of Radiology, G.B. Rossi Hospital, University of Verona, 37134 Verona, Italy; alebele90@hotmail.it (A.B.); giulioriz11@gmail.com (G.R.); drudi.ale@gmail.com (A.D.); greg.aluffi@gmail.com (G.A.); longochiara7@gmail.com (C.L.); alessandro.sarno88@gmail.com (A.S.); 2 Department of Radiology, Ospedale Civile Maggiore, AOUI Verona, 37134 Verona, Italy; riccardo.derobertis@hotmail.it; 3 Department of Oncology, G.B. Rossi Hospital, University of Verona, 37134 Verona, Italy; sara.cingarlini@aovr.veneto.it; 4 Department of Pathology, G.B. Rossi Hospital, University of Verona, 37134 Verona, Italy; paola.capelli@aovr.veneto.it (P.C.); aldo.scarpa@univr.it (A.S.); 5 Department of Surgery, G.B. Rossi Hospital, University of Verona, 37134 Verona, Italy; luca.landoni@aovr.veneto.it (L.L.); claudio.bassi@univr.it (C.B.)

**Keywords:** pancreas, neuroendocrine tumors, computed tomography, texture analysis, tumor burden, grading

## Abstract

Pancreatic neuroendocrine tumors (p-NETs) are a rare group of neoplasms that often present with liver metastases. Histological characteristics, metabolic behavior, and liver tumor burden (LTB) are important prognostic factors. In this study, the usefulness of texture analysis of liver metastases in evaluating the biological aggressiveness of p-NETs was assessed. Fifty-six patients with liver metastases from p-NET were retrospectively enrolled. Qualitative and quantitative CT features of LTB were evaluated. Histogram-derived parameters of liver metastases were calculated and correlated with the tumor grade (G) and ^18^F-fluorodeoxyglucose (^18^F-FDG) standardized uptake value (SUV). Arterial relative enhancement was inversely related with G (−0.37, *p* = 0.006). Different metastatic spread patterns of LTB were not associated with histological grade. Arterial_entropy_ was significantly correlated to G (−0.368, *p* = 0.038) and to Ki67 percentage (−0.421, *p* = 0.018). The ROC curve for the Arterial_entropy_ reported an area under the curve (AUC) of 0.736 (95% confidence interval 0.545–0.928, *p* = 0.035) in the identification of G1–2 tumors. Arterial_uniformity_ values were correlated to G (0.346, *p* = 0.005) and Ki67 levels (0.383, *p* = 0.033). Arterial_entropy_ values were directly correlated with the SUV (0.449, *p* = 0.047) which was inversely correlated with Arterial_uniformity_ (−0.499, *p* = 0.025). Skewness and kurtosis reported no significant correlations. In conclusion, histogram-derived parameters may predict adverse histological features and metabolic behavior of p-NET liver metastases.

## 1. Introduction

Pancreatic neuroendocrine neoplasms represent a rare condition that includes a heterogeneous group of tumors. The WHO classification, recently revised in 2017, divides these tumors into two subgroups, with considerably different prognostic and therapeutic implications based on histological characteristics and Ki67 proliferation index [1,2]. Well-differentiated pancreatic neuroendocrine tumors (p-NETs) are divided into three sub-categories depending on the Ki67 index, reporting a five-year survival up to 85%. The second group is represented by poorly differentiated tumors or pancreatic neuroendocrine carcinoma, for which five-year survival is 9% [3]. The Ki67 percentage is considered the most important prognostic index in gastro-entero-pancreatic neuroendocrine neoplasms [4,5] and is used to define tumor grading (G) and subsequent management. Metastases represent another important prognostic key factor, involving the liver in up to 70% of cases [6,7]. Liver tumor burden (LTB) has been proven to be a good predictor of prognosis and treatment response [5,8,9,10,11,12,13,14]. The metabolic behavior of the tumor related to ^18^F-fluorodeoxyglucose (^18^F-FDG) uptake proved to be another important prognostic factor in p-NETs [15,16]. ^18^F-FDG uptake has been proven to be significantly higher in high-grade than in low-grade neuroendocrine tumors [17]. However, although conventional CT or MR imaging is able to describe the macroscopic characteristics of LTB, it cannot differentiate the histological and metabolic characteristics of p-NETs related to their effective biological aggressiveness. Based on extraction algorithms of the multiple histogram-derived parameters obtainable, texture analysis of biomedical images could represent a valid non-invasive method to easily provide surrogate information on the tumor microenvironment, with a possible prediction of histological and metabolic behavior and then of prognosis and response to treatment [18]. The use of texture analysis techniques is increasingly being studied in biological and medical imaging, having already been proven to be an important aid in the characterization of many tumors including those in the pancreatic field [19,20,21,22,23].

The aim of this study was to evaluate the major qualitative and quantitative imaging features related to metastatic p-NET liver tumor burden, with a particular focus on histogram-derived parameters of the CT texture analysis, in the prediction of histological tumor grade and metabolic activity. 

## 2. Materials and Methods 

### 2.1. Patient Population

We retrospectively enrolled patients with diagnosis of p-NET with liver metastases between April 2010 and December 2016. Patients were selected through a review of radiological, oncological, and pathological databases from our referral center for pancreatic tumors. All procedures performed in studies involving human participants were in accordance with the ethical standards of the corresponding institutional and/or national research committee and with the 1964 Helsinki declaration and its later amendments or comparable ethical standards. This retrospective study was approved by our institutional review board and the requirement for informed consent was waived. The inclusion criteria were: age >18 years; presence of one or more p-NET liver metastases; no previous treatment; availability of the pathological report on the pancreas and/or metastases biopsy; and availability of a contrast-enhanced CT and/or MR performed before the beginning of any oncological treatment. 

### 2.2. Imaging Technique and Evaluation

**Figure 1:**
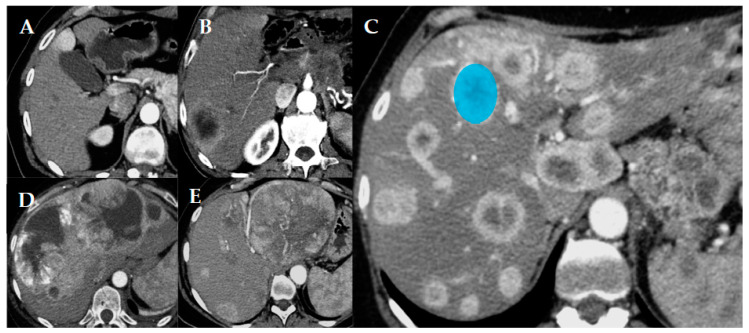
Different patterns of pancreatic neuroendocrine tumor (p-NET) metastatic spread in the liver. Uninodular (**A**), paucinodular (**B**), multinodular (**C**), confluent multinodular (**D**), and bulky (**E**). In image (C) an example of how the volume of interest (VOI; blue circle) was placed when performing the texture analysis is reported.

CT scans acquired in our institute were performed with a multi-slice equipment before and after 1.5 mL/kg intravenous injection of iodine contrast media during arterial phase (15 s after aortic peak) and/or venous phase (60–70 s). Section thickness was 2 mm. The quantitative parameters evaluated were: number of metastases, volume, and major diameter of the largest metastases. Qualitative parameters evaluated were: relative density of metastases in precontrastographic phase, relative enhancement after administration of contrast medium during arterial and venous phase, and pattern of metastatic spread. This pattern (Figure 1) was categorized as follows: uninodular (one lesion), paucinodular (from two to six metastases), multinodular without coalescence of metastases (over six metastases, which remain separated), confluent multinodular, and bulky (huge lesions involving a great portion of the liver, independent of the number of metastases). Unilobar or bilobar involvement of the liver was always evaluated [6]. Other qualitative parameters evaluated were: margins of metastases (sharp or infiltrating), and presence of calcifications, necrosis, and a cystic component. When CT imaging was incomplete or unavailable, the available MR images were consulted for the evaluation of the only missing quantitative (number and dimensions of metastases) and qualitative (relative enhancement and pattern of metastases) characteristics.

### 2.3. Texture Analysis

Texture analysis was performed on the larger vital area of the most representative metastasis, in a single slice, in the arterial (Art) and/or venous (Ven) phase of the CT. The choice of the most representative metastasis on which to place the volume of interest (VOI) for texture analysis (Figure 1) was made arbitrarily according to the following priority criteria in order: absence of artifacts within the lesion; absence of macroscopic cystic components, large areas of necrosis, or coarse calcifications; and a larger lesion available in accordance with previous criteria. Both first- and second-order texture parameters were tested, but only first-order parameters were finally used for the analyses. In particular, the following histogram-derived parameters were included: mean Hounsfield Unit (HU) value, skewness, kurtosis, entropy, and uniformity. Skewness describes the asymmetry of the grey-level distribution in the histogram. Kurtosis reflects the shape of the grey-level distribution (peaked or flat) relative to a normal distribution. Entropy, expressed as log_2_, reflects the randomness of the distribution, while uniformity reflects the uniformity of the distribution. Texture analysis was performed using LIFEx v5.10 software [24] for Mac OS on all the CT exams for which the complete DICOM file was retrievable.

### 2.4. Pathological and PET-CT Evaluation

p-NET diagnosis was confirmed with multiple biopsies on liver metastases and, in some cases, also on the primitive pancreatic tumor. Pathologic features were evaluated according to the 2017 WHO classification system [1], so that grading (G) was established on Ki67 index assessment as follows: G1 for Ki67 index up to 2%, G2 for Ki67 index from 3% to 20%, and G3 for Ki67 >20%. Presence of neuroendocrine cancer (NEC) was also evaluated according to WHO 2017 [1].

All available PET-CT scans performed before any treatment were retrospectively evaluated and the maximum value of standardized ^18^F-FDG uptake of liver metastases (SUV_max_, for convenience abbreviated to SUV) was assessed.

### 2.5. Statistical Analysis

Continuous variables with normal distribution were expressed as mean ± standard deviation, while the median (interquartile range) was used for non-normal distributions. Discrete variables were expressed as percentage of frequency. The Pearson’s test was used to assess correlations between normal distributions, while the Spearman’s test was used for the other correlations. The ANOVA test was used for parametric group comparisons, while the Kruskal–Wallis test was used for non-parametric group comparisons. A chi-squared test was used for the frequency group comparison. ROC curves were used to test the different parameters and to identify an adequate cut-off value to maximize sensitivity and specificity. Binary logistic regression was used to calculate the effect of different parameters on tumor grade and SUV. Level of significance was set at *p* < 0.05. Statistical analysis was performed by using IBM SPSS Statistic v19 software.

## 3. Results

Fifty-six patients were enrolled with a mean age of 57 ± 10 (range 38–86) years, and 34 (60.7%) were males. Three (5.4%) tumors were G1, 40 (71.4%) were G2, and 13 (23.3%) were G3, of which only 4 were NECs. There was no difference in histogram-derived parameters and LTB between G3 pNET and NECs (*p* > 0.05); they were statistically considered together. Median Ki67 was 10 (5–21)%, ranging from a minimum of 2% to a maximum of 80%. Only one pNET was functioning (VIPoma), while the remaining cases were of non-functioning tumors. Only one tumor was non-sporadic (MEN1). SUV values were available only for 35 patients, with a median value of 8 (5.4–12). Population characteristics and main qualitative descriptors of LTB at CT evaluation are collected in Table 1. Texture analysis was performed on 32 patients in the arterial phase and on 22 patients in the venous phase. In other cases, the native CT images were found to be of inadequate quality, not retrievable, unreadable by the software, or corrupt. Mean and median values of histogram-derived parameters are collected in Table 2.

### 3.1. Pattern and Qualitative Descriptors

The metastatic pattern was unilobar in 16 (28.6%) cases and bilobar in 40 (71.4%) cases. The larger the diameter (0.627, *p* < 0.001) and the volume (0.666, *p* < 0.001) of the major lesion, the more the pattern tended to be confluent and bulky. A direct correlation was found with arterial relative enhancement, with a tendency for hypervascular metastases to be more confluent and bulky (0.394, *p* = 0.003), whereas the relative density in the precontrastographic, venous, and late phases was not correlated with the pattern. Multinodular patterns were associated with a greater presence of necrosis (0.498, *p* < 0.001) and a greater presence of cystic components (0.276, *p* = 0.04). The greater the number of metastases, the more likely there was to be a necrotic component (0.394, *p* = 0.003), and the more the metastases tended to be hypodense in the precontrastographic phase (−0.285, *p* = 0.043) and hyperdense in the arterial phase (0.341, *p* = 0.012).

### 3.2. Tumor Grade and Ki67

#### 3.2.1. Qualitative Descriptors

The number of metastases tended to be lower as the tumor grade increased (−0.275, *p* = 0.04); however, Ki67 did not correlate with the overall number of metastases in the liver (*p* = 0.326). On the other hand, there was no correlation between G or Ki67 and the size of the largest lesion. The relative venous enhancement did not correlate with G or Ki67, whereas G was significantly inversely correlated to relative arterial enhancement (−0.37, *p* = 0.006); therefore, with the increase of G the metastases tended to be more hypovascular. In particular, the risk of G3 on biopsy was much lower when the metastases were hypervascular as compared to hypovascular (OR 0.18, 95% confidence interval (CI) 0.05–0.69, *p* = 0.012). The metastatic pattern was not related to G (*p* = 0.360) nor to Ki67 (*p* = 0.575). Unilobar or bilobar involvement of the liver was not correlated to tumor grade (*p* = 0.486). No difference was observed in the type of margins (infiltrating or sharp) in the different groups of G, nor for the presence of necrosis, calcification, or a cystic component of the metastases. ROC curves showed no significant results (*p* > 0.05) for the number of metastases, the diameter of the largest lesion, and the spread pattern in the identification of G1–2 vs. G3 pNETs. Therefore, different metastatic patterns were not associated with a prediction of histological tumor grade.

#### 3.2.2. Histogram-Derived Parameters

**Figure 2:**
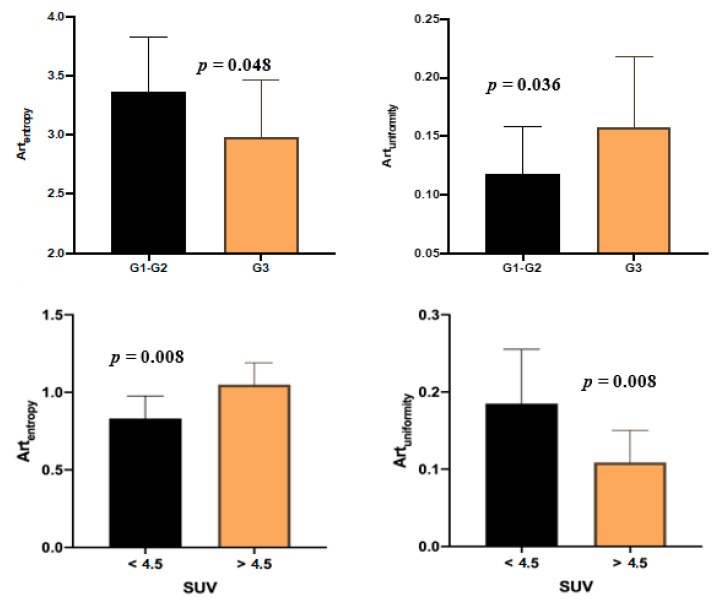
Comparison bar graph of the mean values of Art_entropy_ and Art_uniformity_ in the different groups for tumor grade (G) and ^18^F-FDG standardized uptake values (SUV).

**Figure 3:**
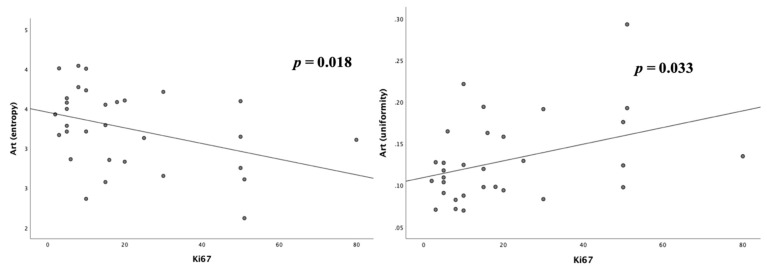
Scatter plots of correlation between Ki67 levels and Art_entropy_ (**left**) and Art_uniformity_ (**right**).

**Figure 4:**
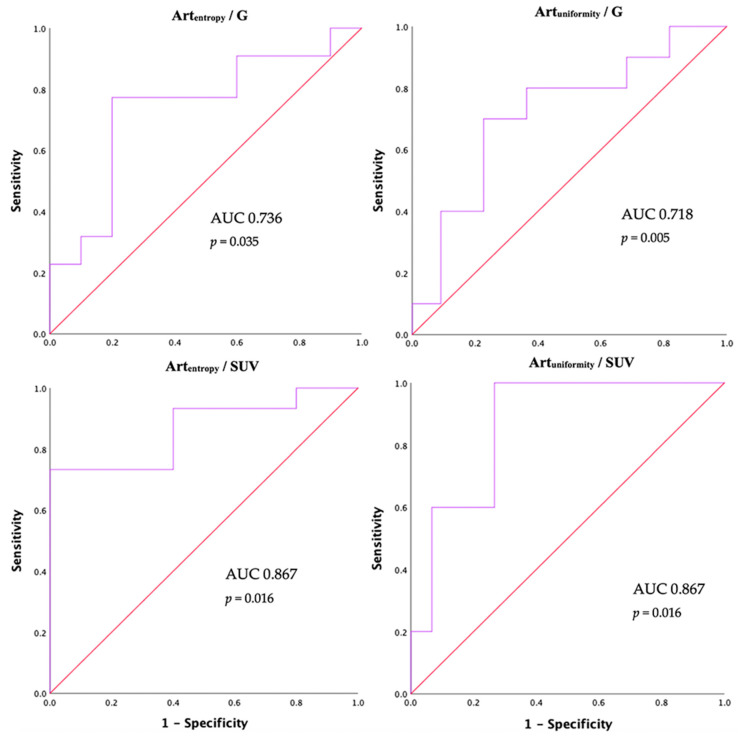
ROC curves for Artentropy and Artuniformity in the identification of the group with respect to tumor grade (G) and 18F-FDG standardized uptake values (SUV).

Art_entropy_ was significantly related to the tumor grade (−0.368, *p* = 0.038) and to the percentage of Ki67 at biopsy (−0.421, *p* = 0.018,). The mean Art_entropy_ was different in the G1–2 vs. G3 groups (3.37 ± 0.46 vs. 2.99 ± 0.47, *p* = 0.048, Figure 2 and Figure 3). The ROC curve test for the Art_entropy_ (Table 3) showed an area under the curve (AUC) of 0.736 (95% CI 0.545–0.928, *p* = 0.035) in the identification of the G1–2 tumors (Figure 4). The 3.16 Art_entropy_ cut-off reported 77.3% sensitivity and 80% specificity in identifying G1–2. For Art_entropy_ values beyond this cut-off, a considerable reduction in the risk of being G3 was observed (OR 0.07, 95% CI 0.012–0.464, *p* = 0.005). 

The distribution above and below this cut-off of Art_entropy_ with respect to G1–2 vs. G3 was significantly different in the chi-squared test (*p* = 0.005). Specifically, 17 (77.3%) G1–2 tumors were above the cut-off, while only 2 (20%) G3 tumors were above. Eight (61.5%) tumors under the Art_entropy_ cut-off were G3, while only two (10%) tumors above the cut-off were G3. The median Ki67 in lesions with Art_entropy_ below and above the identified cut-off was significantly different (27 (15–51) vs. 8 (5–15), *p* = 0.001). Art_kurtosis_ and Art_skewness_ did not correlate with tumor grade or Ki67 percentage. The ROC curve test did not report satisfactory results for these last two first-order descriptors. The Art_uniformity_ values were related to the tumor grade (0.346, *p* = 0.005) and Ki67 levels (0.383, *p* = 0.033, Figure 2). The ROC curve for this descriptor reported an AUC of 0.718 (95% CI 0.522–0.914, *p* = 0.05) in identifying the G3 tumors (Figure 4). 

The Art_uniformity_ cut-off of 0.122 showed 80% sensitivity and 64% specificity in identifying G3 tumors (Table 3). On logistic regression, the risk of having a G3 tumor was increased beyond this cut-off (OR 7, 95% CI 1.18–41.4, *p* = 0.032). Categorizing the sample with respect to this Art_uniformity_ cut-off it was observed that the frequencies were different in the different groups of G with the chi-squared test (*p* = 0.05), with two (20%) G3 tumors under this Art_uniformity_ cut-off and eight (80%) G3 above. Only two (12.5%) tumors above this cut-off were of G3. The median of Ki67 below and above the Art_uniformity_ cut-off identified at the ROC curve was significantly different (27 (15–51) vs. 8 (5–15), *p* = 0.034).

Art_entropy_ and Art_uniformity_ were inversely related (−0.968, *p* < 0.001) and a strong direct correlation between Art_entropy_ and Ven_entropy_ was also observed (0.821, *p* < 0.001). Ven_entropy_, however, was not correlated to the tumor grade nor to the percentage of Ki67 as the arterial counterpart. In the same way, Ven_uniformity_ and Ven_kurtosis_ do not correlate with the tumor grade nor with Ki67. Only one parameter in venous phase was shown to correlate with the percentage of Ki67, and that was the Ven_skewness_ (0.46, *p* = 0.031). However, on the ROC curve test, this was not confirmed as a reliable parameter to identify the G3 tumor (*p* = 0.192). No significant differences were observed in the mean values of Ven_skewness_ in the G1–2 vs. G3 group (*p* = 0.335). The hypervascularization of liver metastases was correlated to higher Art_entropy_ (0.461, *p* = 0.008), lower Art_uniformity_ (0.477, *p* = 0.006) and Art_kurtosis_ (−0.5, *p* = 0.004), and lower presence of macroscopic necrosis at CT (−0.449, *p* = 0.001).

### 3.3. 18F-FDG Standardized Uptake Value

#### 3.3.1. Qualitative Descriptors

SUV values of the metastases were not correlated to the tumor grade (*p* = 0.703) nor to Ki67 percentage and they did not correlate with the metastatic pattern. The SUV of the lesions was directly correlated to the size of the biggest metastasis (0.442, *p* = 0.008) and to its volume (0.421, *p* = 0.012), but it was not dependent on the overall number of liver metastases. The SUV directly correlated to the relative enhancement in the arterial phase (0.359, *p* = 0.037) and inversely correlated with the relative density in the pre-contrastographic phase (−0.459, *p* = 0.009). The relative density in the venous phase was not correlated to the maximum SUV of the lesions. A higher SUV was observed in the presence of liver lesions with a necrotic component as compared to those where the necrotic component was absent (9.5 (7.1–13) vs. 3.8 (2.2–90), *p* = 0.017).

#### 3.3.2. Histogram-Derived Parameters

There was no correlation between maximum SUV values of metastases and mean densities of the target lesion chosen for the texture analysis in the arterial and venous phases. Art_entropy_ values were correlated directly with the maximum SUV (0.449, *p* = 0.047); therefore, the higher the Art_entropy_ the higher the SUV. The sample was categorized into ^18^F-FDG hypocaptant (<4.5) and hypercaptant (>4.5) SUV values to perform a group comparison. The mean Art_entropy_ was different in the two SUV <4.5 vs. SUV >4.5 groups (2.76 ± 0.49 vs. 3.49 ± 0.47, *p* = 0.008). Art_entropy_ reported an AUC of 0.867 (95% CI 0.704–1, *p* = 0.016) in identifying SUV >4.5 (Figure 4). The Art_entropy_ cut-off of 2.6813 reported 93.3% sensitivity and 60% specificity for identifying a maximum SUV >4.5 (Table 4). An inverse correlation between SUV and Art_uniformity_ was observed (−0.499, *p* = 0.025), for which the mean was statistically different in the presence of SUV <4.5 compared to SUV >4.5 (0.18 ± 0.07 vs. 0.11 ± 0.04, *p* = 0.008). Art_uniformity_ showed an AUC of 0.867 (95% CI 0.704–1, *p* = 0.016) in the detection of metastases with SUV <4.5. The Art_uniformity_ cut-off of 0.1224 reported 80% sensitivity and 73.3% specificity to identify the presence of SUV <4.5 in liver metastases. Art_kurtosis_ also correlated inversely with the SUV of liver metastases (−0.529, *p* = 0.016), unlike the Art_skewness_ which did not report significant correlations. The ROC curve test did not produce satisfactory results for these last two first-order descriptors. No correlation was observed between Ven_entropy_, Ven_uniformity_, Ven_kurtosis_, nor Ven_skewness_ with SUV values of liver metastases.

## 4. Discussion

Metastatic disease is present in 80% of cases of p-NETs at diagnosis, and represents a key prognostic factor to consider in therapeutic management. The liver is the most involved organ in neoplastic diffusion [6,7]. Although many studies have shown that prognosis of p-NETs with liver metastases varies according to the extent of LTB [5,8,9,10] and that LTB is predictive of treatment response [11,12,13,14], the European Neuroendocrine Tumor Society (ENETS) and American Joint Committee on Cancer (AJCC) staging systems do not consider metastatic involvement per organ, nor the extension of liver involvement [1]. Some CT and MR findings are effective in predicting the aggressiveness and grade of the tumor, for example as arterial phase hypovascularization, major vessel invasion, or size [25,26,27]. In particular, many studies in the literature have described the hypoenhancement of p-NETs tumors as a strong predictor of a high-grade tumor [28,29,30,31]. Our study reported analogous results on this latter parameter, with the hypovascularization of liver metastases being one of the best LTB descriptors in the prediction of high tumor grade. No significant difference in the other LTB descriptors (e.g., shape of the margins, calcifications) was observed between the different tumor grades, with the exception of a slight correlation with the overall number of liver metastases which, however, did not correlate with the continuous Ki67 value. Similar results were observed by Denecke et al. [31] in their study on G1 and G2 pNET liver metastases, where hypoenhancement was a negative prognostic factor for early tumor progression, in contrast to the number of metastases. In our study, texture analysis provided better results in the prediction of tumor grade than many common descriptors of LTB, identifying some histogram-derived parameters with significantly different distributions in the various G groups. This is in line with what was found by Choi et al. [32] in their study on prediction of G1 vs G2–3 pNETs, where diagnostic performance of texture analysis was superior to CT findings. 

The correlation between entropy values and different tumor grades of p-NETs has already been a matter of study [26,30,32,33]. In these studies, entropy was found to be an excellent predictor of the tumor grade when comparing G1 vs. G2–3 [26,32]. However, the comparison was made mainly between G1 and G2–3, as the G3 tumors represented a minority within the study population. In the study by Guo et al. [30] which mainly focused on G1–2 vs. G3 neuroendocrine tumor of the pancreas, a significant inverse correlation was observed between entropy and tumor grade, with sensitivity and specificity values comparable to those found in our study on texture analysis performance in pNETs liver metastases. 

The fact that entropy, which is an index of inhomogeneity, is lower in high-grade tumors could be seen as a contradiction considering that a greater degree of heterogeneity in the most aggressive lesions could be expected. However, we evaluated the entropy in the arterial phase, and although pNETs are classically hypervascular, it has been proven in the present study that most aggressive lesions result hypovascular. Therefore, G3 tumors may have lower Art_entropy_ and higher Art_uniformity_, being homogeneously hypovascularized tumors in the arterial phase as compared to G1 and G2 tumors which, being more frequently hypervascular, demonstrate a certain degree of inhomogeneity when the iodine contrast is in the early stage of filling. Necrosis and fibrosis would play a role. Another possible explanation for the lower entropy observed in high-grade tumors may lie in the vascular permeability. In a study by Ng et al. [34] on texture analysis of the CT venous phase in colorectal cancer it was observed that low-entropy tumors were associated with poorer prognosis. The authors attributed this to the greater extravascular distribution of the contrast medium, with consequent reduction of the contrast resolution between vessels and parenchyma and then reduction of heterogeneity and entropy. In our study, a greater vascular permeability of high-grade pNETs combined with higher cell packing could explain the lower entropy observed in the more aggressive tumors. 

The choice to test both the G and the Ki67 value in parallel derives from the fact that the cut-off to differentiate G1 from G2 p-NETs from a prognostic point of view is controversial [3,35], particularly for G2 sub-category which includes a too-wide range of tumors, ranging from benign tumors, which benefit from more conservative management, to malignant tumors which have a much more aggressive behavior. The correlation found in our study between Art_entropy_ and Art_uniformity_ not only with the G but also with the continuous value of Ki67 would make the results valid in the future if the categorization cut-off were to be redefined.

In our study Art_entropy_ was directly correlated with SUV values of liver metastases, which were inversely correlated with Art_kurtosis_. Higher SUV values were measured in the presence of higher Art_entropy_ which, however, was indicative of a lower tumor grade. This apparently contradicts the literature reporting that metastatic pNETs with higher ^18^F-FDG uptake are more frequently of high grade [16,17] and are associated with a worse prognosis, both in terms of progression-free survival and overall survival [15]. We found no correlation between SUV and tumor grade or Ki67. In our study, low SUV values were reported for hypovascularized tumors with low heterogenicity in the arterial phase. However, the Art_entropy_ cut-offs identified for predictivity of tumor grade and SUV were different, at 3.16 and 2.68, respectively. It could be interpreted that for higher Art_entropy_ values there could be low-grade pNET metastases but with a positive SUV, for lower Art_entropy_ values there could be high-grade metastases but with a negative SUV, and for intermediate Art_entropy_ values there could be high-grade metastases with a high SUV. This explanation based on a nonlinear correlation between the extreme Art_entropy_ values with the grade and the SUV is purely technical, and it must be taken in consideration that the relatively small study population could be reason for this result. Moreover, important changes in tumor composition affecting tumor vascularization could have a great impact on the results. Still, it should be considered that the maximum SUV in our study did not always refer to the target lesion for texture analysis but to the highest SUV value observed in all liver metastases, and this must be taken in consideration given the extreme heterogeneity of neuroendocrine tumors both at imaging and biologically, at times with the coexistence of well-differentiated and poorly differentiated pNETs clones in the same patient. Therefore, on the one hand the presence of high levels of Art_entropy_ should be reassuring, as it is associated with a lower tumor grade; on the other it should raise attention in order to search for any other possible lesion with a higher metabolism and with high risk of progression.

Texture analysis proved to be a useful method to support the diagnosis of histological and metabolic characteristics of pNET liver metastases. Currently, it cannot replace biopsy at diagnosis, which remains essential for correct therapeutic and oncological planning. In the near future, the computerized quantitative analysis of the histogram-derived parameters could instead be of great help in identifying the most at-risk metastases that the radiologist’s eye cannot identify, for which a biopsy may be indicated in search of higher-grade metastases. Since the correlation between texture analysis and tumor grade has been proven, this result could also be applied in follow-up to depict high-grade progression and avoid re-biopsies. Moreover, we are entering the era of artificial intelligence-assisted medicine. In this sense, the results of our study may eventually be used to develop automatic or semi-automatic software-based tools for the identification and correct characterization of the pNETs.

The main limitations of this study are its retrospective nature and relatively low sample size, responsible for the amplitude of some calculated confidence intervals. Being a retrospective study, it was not always possible to find all the data for each patient, and texture analysis was not possible for some patients. For the same reason, the CT images of few patients coming from different hospitals used for texture analysis were not always acquired with the same scanner and with the exact same phases, even if a conformity assessment was done. Nevertheless, the significance obtained in this study despite the non-standardization of the CT protocol for all patients makes the results more applicable in clinical practice, where each institution uses different scanners with subtle differences in acquisition protocols. Finally, texture analysis was always done on the same lesion on which the biopsies were performed, assuming that all liver metastases had the same tumor grade seen on biopsy. Further prospective studies on a larger study population will be needed in the future to confirm what has been observed and to test the histogram-derived parameters of liver pNET metastases in the prediction of prognosis of the patients and overall survival. 

## 5. Conclusions

Texture analysis of liver metastases from p-NETs performed on contrast-enhanced CT images can be a useful non-invasive method in the prediction of histological tumor grade and metabolic behavior supporting the other common descriptors of liver tumor burden. Histogram-derived parameters could help to identify the highest-grade liver metastases to be biopsied and to raise the suspicion of high-grade progression at follow-up.

## Figures and Tables

**Table 1:** Population characteristics and qualitative descriptors of liver tumor burden.

Parameter (*n* = 56)	Value (*n*)	Parameter (*n* = 56)	Value (*n*)
Age (years)	57 ± 10	Ki67 (%)	10 (5–21) *
Sex (males)	60.7	SUV ^1^	8 (5.4–12) *
Tumor grade (%) 1 2 3	5.4 (3) 71.4 (40) 23.3 (13)	Pre-contrast (%) Hypodense Isodense Hyperdense	92.2 (52) 5.8 (3) 2 (1)
Pattern (%) Uninodular Paucinodular Multinodular Confluent multinodular Bulky	7.1 (4) 17.9 (10) 55.4 (31) 10.7 (6) 8.9 (5)	Arterial phase (%) Hypodense Hyperdense	25.9 (15) 74.1 (41)
		Venous phase (%) Hypodense Isodense Hyperdense	81.5 (46) 7.4 (4) 11.1 (6)
Number of metastases	14 (4–43) *	Calcifications (%)	7.1 (4)
Greater metastasis (mm)	39 (22–65) *	Cystic (%)	10.7 (6)
Sharp margins (%)	85.7 (48)	Necrosis (%)	64.3 (36)
Sporadic (%)	98.2 (55)	Functioning (%)	1.8 (1)

^1^ SUV: maximum standardized ^18^F-fluorodeoxyglucose (^18^F-FDG) uptake value. * the values in brackets refer to the interquartile range.

**Table 2:** First-order texture analysis parameters in the arterial and venous phase with ANOVA or Kruskal–Wallis tests for the comparisons between different tumor grade (G) and SUV groups.

	Total	Tumor Grade			SUV ^1^		
		1–2	3	*p*	<4.5	>4.5	*p*
Art_HUmean_	77 (65–95)	83 (51–95)	67 (53–81)	0.569	113 (67–159)	83 (62–94)	0.760
Art_entropy_	3.25 ± 0.49	3.37 ± 0.46	2.99 ± 0.47	0.038	2.76 ± 0.49	3.49 ± 0.47	0.008
Art_uniformity_	0.13 ± 0.05	0.12 ± 0.04	0.16 ± 0.06	0.036	0.18 ± 0.07	0.11 ± 0.04	0.008
Art_kurtosis_	2.94 (2.66–3.43)	2.86 (2.54–3.37)	3.07 (2.72–3.62)	0.272	3.51 (3.07–3.96)	2.78 (2.48-2.99)	0.106
Art_skewness_	−0.04 (−0.18–0.14)	−0.03 (−0.43–0.14)	−0.10 (−0.15–−0.02)	0.776	−0.14 (−0.43–0.14)	−0.06 (−0.47–−0.01)	0.827
Ven_HUmean_	86 ± 19	86 ± 18	87 ± 23	0.938	104 ± 23	88 ± 15	0.147
Ven_entropy_	3.15 ± 0.4	3.23 ± 0.41	2.98 ± 0.33	0.172	2.98 ± 0.70	3.28 ± 0.39	0.324
Ven_uniformity_	0.14 ± 0.04	0.13 ± 0.036	0.15 ± 0.04	0.184	0.16 ± 0.07	0.13 ± 0.04	0.210
Ven_kurtosis_	3.04 (2.68–3.78)	3.06 (2.72–3.78)	3.05 (2.52–3.76)	0.916	3.40 (3.05–3.76)	2.89 (2.59–3.45)	1.000
Ven_skewness_	−0.11 ± 0.47	−0.18 ± 0.45	0.03 ± 0.51	0.335	0.16 ± 0.74	−0.27 ± 0.29	0.423

^1^ SUV: maximum standardized ^18^F-FDG uptake value.

**Table 3:** One-way ANOVA and ROC tests on Art_entropy_ and Art_uniformity_ for the identification of high tumor grade.

	Art_entropy_	Art_uniformity_
Tumor grade 1–2	3.37 ± 0.46	0.12 ± 0.04
Tumor grade 3	2.99 ± 0.47	0.16 ± 0.06
AUC (95% CI) *	0.736 (0.545–0.928)	0.718 (0.522–0.914)
Cut-off	3.16	0.12
Sensitivity (%)	77.3	80
Specificity (%)	80	64

* Area under the curve (95% confidence interval).

**Table 4:** One-way ANOVA and ROC tests on Art_entropy_ and Art_uniformity_ for the identification of high SUV values.

	Art_entropy_	Art_uniformity_
SUV ^1^ <4.5	2.76 ± 0.49	0.18 ± 0.07
SUV ^1^ >4.5	3.49 ± 0.47	0.11 ± 0.04
AUC (95% CI) *	0.867 (0.704–1)	0.867 (0.704–1)
Cut-off	2.68	0.12
Sensitivity (%)	93.3	80
Specificity (%)	60	73.3

* Area under the curve (95% confidence interval). ^1^ SUV: maximum standardized ^18^F-FDG uptake value.
